# Toward More Translational Tumor Models: Breast dECM-Based 3D Systems Capture Native Microenvironmental Cues

**DOI:** 10.3390/bioengineering13060712

**Published:** 2026-06-21

**Authors:** Katherine L. Hebert, Jonathan J. Savoie, Mackenzie L. Hawes, Britney Nguyen, Madison Lee, Marcus A. Moody, Sophie R. Dietrich, Thomas Cheng, Van H. Barnes, Bridgette M. Collins-Burow, Alison A. Smith, Frank H. Lau, W. Todd Monroe, Matthew E. Burow, Elizabeth C. Martin, Jorge A. Belgodere

**Affiliations:** 1 Tulane Department of Medicine, Section of Hematology & Oncology, Tulane University Health Science Center, New Orleans, LA 70112, USA; khebert6@tulane.edu (K.L.H.); mhawes@tulane.edu (M.L.H.); mmoody1@tulane.edu (M.A.M.); sdietrich@tulane.edu (S.R.D.); tcheng1@tulane.edu (T.C.); vbarnes2@tulane.edu (V.H.B.); bcollin1@tulane.edu (B.M.C.-B.); mburow@tulane.edu (M.E.B.); 2 Department of Biological and Agricultural Engineering, Louisiana State University and Agricultural Center, Baton Rouge, LA 70803, USA; jsavo12@lsu.edu (J.J.S.); bnguyen11@tulane.edu (B.N.); mlee25@lsuhsc.edu (M.L.); tmonroe@lsu.edu (W.T.M.); 3 Tulane Cancer Center, Tulane University, New Orleans, LA 70112, USA; 4 Department of Clinical Surgery, Louisiana State University School of Medicine, New Orleans, LA 70112, USA; asmi60@lsuhsc.edu; 5 Department of Medicine, Tulane University, New Orleans, LA 70112, USA; frank.lau@keliomics.com; 6 Keliomics, Inc., Portland, OR 97239, USA

**Keywords:** decellularized tumor, extracellular matrix, triple-negative breast cancer, 3D tumor model, breast adipose, new approach methods

## Abstract

Current 3D tumor models for aggressive breast cancers inadequately recapitulate the native tumor microenvironment (TME), leading to poor translational potential. There is a critical need for models capable of mimicking the unique biochemical signals present in the TME. To address this gap, breast tissue and a patient-derived xenograft tumor were decellularized and processed to produce breast tissue- and tumor-specific decellularized extracellular matrices (dECM). Histology confirmed complete cellular removal while maintaining the ECM. Further, DNA content was significantly reduced while ECM composition (POSTN, COLI, FN1) was retained. Breast dECM was incorporated (0, 5, 10, 20, and 50 µg/mL) with triple-negative breast cancer cell lines to generate spheroids. Imaging and histology demonstrated that cells in low dECM (5 and 10 µg/mL) formed compact singular spheres, while higher dECM concentrations (20 and 50 µg/mL) resulted in cells concentrated on the outer edge of the sphere and irregular sphere circularity. RNA-sequencing of MDA-MB-231 dECM spheres demonstrated that gene changes were mediated by both the inclusion of dECM and its composition. High-density tumor dECM upregulated genes associated with metastasis, while high-density breast dECM enhanced tumor suppressors and anti-metastasis genes. These findings indicate that dECM provides physiological cues in 3D tumor models by incorporating TME.

## 1. Introduction

Breast cancer is a highly heterogeneous disease reflecting both intrinsic and extrinsic heterogeneity [1,2]. This diversity in breast tumors arises from genetic mutations, epigenetic alterations, and dynamic interactions within the tumor microenvironment (TME) [1,3]. Consequently, these changes have led to variations in proliferative capacity, metastatic potential, and therapeutic responsiveness sensitivity [4,5,6]. Tumor heterogeneity and inter-patient variations often limit the success of predicting prognosis and therapeutic interventions, which pose a significant challenge in cancer modeling [7]. Understanding and addressing tumor heterogeneity is critical to developing personalized therapies and improving clinical outcomes in breast cancer patients [7]. Despite advances in tumor modeling, accurately recapitulating breast cancer complexity in vitro remains a major barrier for modeling disease progression and therapeutic response [8,9]. This is especially critical for triple-negative breast cancer (TNBC), where roughly 20% of patients demonstrate therapy resistance, and prior works have highlighted a lack of in vitro models to mimic the TNBC tissue environment [10].

Three-dimensional (3D) tumor models have emerged as valuable tools to recapitulate the complexities of tumor heterogeneity, offering an advantage over 2D cell culture systems [11]. Breast cancer cells grown using 3D platforms more closely mimic the complexities of in vivo tumors compared to 2D, including tumor morphology and topography [12], upregulation of pro-angiogenic proteins [12], inclusion of biochemical signals, cell–cell and cell–matrix interactions [13], nutrient gradients [14], and the incorporation of extracellular matrix (ECM)-related components [15]. By providing a more accurate representation of the TME, a better assessment of cancer progression offers new insights into target identification and evaluation of new therapeutic options [16], in a more physiologically relevant context [17].

Approaches for 3D modeling in cancer research can be categorized into scaffold-based and scaffold-free methods. Scaffold-free 3D modeling entails cultured cells assembled into 3D structures without external scaffold material [18]. Traditional spheroids, for example, comprise cells that self-assemble and interact with neighboring cells to form 3D-like structures. This type of 3D modeling is beneficial as they allow for more accurate cell–cell interactions, spatial organization, and physiological responses, making them valuable tool for various types of cancer studies [19]. However, the main disadvantage of scaffold-free spheroids is the lack of mechanical support, shape control, and reproducibility compared to scaffold-based 3D modeling [20]. ECM-based modeling can provide a unique advantage to non-scaffold spheroids through an enhanced 3D support structure, presenting a platform for cells to attach, proliferate, and interact with their surroundings [21]. ECM-derived 3D structures allow for more accurate evaluations of tissue-specific processes in cancer progression, including the microenvironmental influence on cellular mechano-transduction, cell morphology, proliferation, and gene expression [16].

In tissue engineering applications, decellularized tissue can be utilized to generate platforms for organ and tissue modeling to overcome current in vitro limitations [22,23]. Tissue decellularization is a processing technique to remove cellular components from tissues or organs while preserving the complex architecture, composition, and biological activity of the ECM [24]. The ECM serves as a biological scaffold, or a 3D framework, that retains native tissue proteins, including structural proteins such as collagens, elastin, fibronectin, laminin, and other matricellular proteins [25]. Protein retention maintains mechanical integrity and the biochemical signals essential for cellular attachment, proliferation, and differentiation [24,26]. For example, using decellularized tissue to generate platforms for 3D breast cancer modeling can play a pivotal role in understanding cancer biology and enabling the development of effective therapeutics [27]. Decellularized scaffolds offer a unique advantage by providing a biologically relevant microenvironment that synthetic materials often fail to replicate [28].

Despite a concerted effort to shift away from traditional 2D modeling approaches, for pre-clinical studies in breast cancer, current 3D models lack the incorporation of native ECM and associated cell–ECM-driven biological changes. This study aims to evaluate the impact of breast adipose decellularized ECM (dECM) and tumor dECM on the development and transcriptome of 3D tumor spheroids with the following objectives: (1) to optimize decellularization of native breast adipose tissue to confirm complete removal of cellular and lipid components and retention of ECM proteins (COL1A1, FN, and POSTN); (2) to determine the impact dECM plays in TNBC spheroid formation and organization; and (3) a to provide proof-of-concept evaluation that dECM can alter the transcriptome of TNBC spheroids.

## 2. Materials and Methods

### 2.1. Cell Culture

TU-BcX-4IC, a metaplastic TNBC cell line, was previously established in our lab [29], and MDA-MB-231, a non-metaplastic TNBC cell lines, (ATCC, Manassas, VA, USA) were cultured in Dulbecco’s Modified Eagle Medium (DMEM) (Gibco, Waltham, MA, USA) supplemented with 10% HyClone Cosmic Calf Serum (Cytiva, Wilmington, DE, USA), 1% minimal essential amino acids (Gibco, Waltham, MA, USA), 1% non-essential amino acids (Gibco, Waltham, MA, USA), 1% antibiotic/anti-mycotic (Gibco, Waltham, MA, USA), and insulin (Gibco, Waltham, MA, USA) under mycoplasma-free conditions at 37 °C and 5%* v*/*v* CO_2_.

### 2.2. Breast Adipose Tissue

Human breast tissue was collected from normally discarded breast specimens generated during elective surgical procedures. Donor tissue came from female patients with an average age of 34.7 ± 13.12 and body mass index of 30.5 ± 2.97 (Table S1).

### 2.3. TNBC Patient-Derived Xenograft (PDX) Generation

The TU-BcX-4IC primary TNBC metaplastic tumor was originally collected from a mastectomy biospecimen and described in our previously published works [29]. TU-BcX-4IC PDX propagation was performed as previously described [29] in SCID/Beige mice between 8 and 12 weeks of age at implantation. In brief, TU-BcX-4IC PDX samples were approximately 3 × 3 mm^3^ upon implantation into mice. Surgical conditions are outlined in [30]. Once the tumor became palpable, measurements were recorded once weekly with calipers. Tumors were allowed to grow to a maximum size of 20 mm (or 2 cm^3^) or until mice demonstrated signs of morbidity and were euthanized before the maximum allotted size was reached. Tumors were then processed to create tumor dECM. Research was conducted under Institutional Animal Care and Use Committee protocol 1515 and approved by the Institutional Review Board at Tulane University.

### 2.4. Tissue Decellularization (dECM) and Homogenization

Breast adipose tissue and PDX were decellularized using a modified freeze–thaw method (−80 °C to 37 °C), based on modified previously described protocols by [31,32]. Briefly, tissues were cut into small pieces (~4–5 g), frozen in −80 in a freezing buffer containing 10 mM Tris (pH 8.0) and 5 mM ethylenediaminetetraacetic acid (EDTA), and then thawed. Samples were then placed in Enzymatic Digestion Solution #1 (0.25% trypsin (ThermoFisher Scientific, Waltham, MA, USA)/0.1% EDTA (ThermoFisher Scientific, Waltham, MA, USA) and placed on an orbital shaker. After 24 h, the solution was replaced with a lipid extraction solution, isopropyl alcohol (IPA, ThermoFisher Scientific, Waltham, MA, USA), and 1% anti-anti. After 24 h, samples were then incubated in Enzymatic Digestion Solution #2 (55 mM Na_2_HPO_4_, 17 mM KH_2_PO_4_, 4.9 mM MgSO_4_·7H_2_O, 150 U/mL DNase Type II [bovine pancreas], 0.125 mg/mL RNase Type III A [bovine pancreas], and 20 U/mL Lipase Type VI-S [porcine pancreas]) for 24 h, followed by a second lipid extraction step. Following decellularization, samples were rinsed and stored in PBS anti-anti (Gibco, Waltham, MA, USA) at 4 °C until lyophilization. Tissues were then frozen at −80 °C, lyophilized using a Labconco FreeZone 4.5 L Benchtop Freeze Dryer (Labconco Corporation, Kansas City, MO, USA), and then cryogenically milled using a Cole-Parmer SamplePrep CG-400-115 Freezer/Mill^®^ Large Cryogenic Grinder (115 VAC, 60 Hz; Cole-Parmer, Vernon Hills, IL, USA). The resulting homogenized dECM powder was collected and stored at −20 °C.

### 2.5. Tissue Composition Characterization

Native breast adipose tissue samples were processed to quantify water, lipid, and protein fractions by weight. Fresh tissue (4–10 g) was weighed and lyophilized for 24 h to determine water content. Dehydrated samples were then transferred to 50 mL conical tubes and submerged in 99% IPA to extract lipids, with gentle agitation overnight at room temperature. IPA was replaced the following day, and washing continued for up to two additional overnight cycles until tissue appeared white. Samples were subsequently transferred to pre-weighed boats, air-dried to remove residual IPA, and re-weighed. Water content was calculated as the mass lost after lyophilization, lipid content as the mass lost following IPA extraction, and the remaining mass was attributed to protein-rich connective tissue (ECM) fraction.

### 2.6. Tissue Histology

To assess changes in tissue morphology and confirm decellularization, native and decellularized breast tissues were fixed in 10% formalin (Sigma-Aldrich, St. Louis, MO, USA), paraffin-embedded, sectioned into 5 mm sections, and stained for hematoxylin and eosin (H&E) and Masson’s trichrome (MTC) by the Histology Core at Tulane University School of Medicine. Slides were then imaged, and representative images were presented. TU-BcX-4IC PDX decellularization confirmation was performed as previously described [33].

### 2.7. DNA Quantification

DNA was extracted from native breast and decellularized breast tissue using a DNEasy kit (Qiagen Laboratories, Hilden, Germany), following a modified extraction protocol [34]. Briefly, 200 mg of tissue was immersed in 400 µL of Qiagen ATL lysis buffer, with 20 µL of Proteinase K solution, and incubated overnight. 200 µL of ATL buffer was added, tubes were centrifuged, and the aqueous layer was transferred to a new tube with buffer ATL and molecular grade ethanol (ThermoFisher Scientific, Waltham, MA, USA). Samples were transferred to a DNA micro column for successive buffer washes, centrifugations, and discarding the flow-through. DNA was eluted in 100 µL of water. DNA content was measured using a NanoDrop 1000 spectrophotometer (ThermoFisher Scientific, Waltham, MA, USA). DNA gel electrophoresis was performed to further confirm the loss of DNA. Gels were run using a 1 kbp ladder, 1% agarose gel (ThermoFisher Scientific, Waltham, MA, USA), a run time of 1 h and 30 min, and an exposure time of 25 ms.

### 2.8. Protein Extraction and Quantification

Protein from both native and decellularized primary breast adipose tissue and PDX tumor were homogenized and lysed in RIPA lysis and extraction buffer (ThermoFisher Scientific, Waltham, MA, USA) in addition to Halt^TM^ Protease and Phosphatase Inhibitor Cocktail (100×) (ThermoFisher Scientific, Waltham, MA, USA). The samples were incubated for 1 h on ice and centrifuged at 14,000× *g* for 15 min at 4 °C. The supernatant was collected in a new tube and stored at −80 °C until use. RIPA-isolated protein concentration was measured via the Pierce^TM^ BCA Protein Assay Kit (ThermoFisher Scientific, Waltham, MA, USA). Protein concentration was normalized across samples, and concentrations of POSTN, COL1A1, and FN1 were measured via the Human Periostin/OSF-2 DuoSet ELISA kit (R&D Systems, Minneapolis, MN, USA), Human Pro-Collagen 1 alpha 1 DuoSet ELISA kit (R&D Systems, Minneapolis, MN, USA), and the Human Fibronectin DuoSet ELISA kit (R&D Systems, Minneapolis, MN, USA).

### 2.9. dECM Spheroid Fabrication

Tumor spheroid formation was modified from methods previously published by others [35]. Briefly, MDA-MB-231 and Tu-BcX-4IC cells were seeded in Nunclon Sphera 96-well U-bottom plates (Thermo Fisher, Waltham, MA, USA) with increasing concentrations of dECM (0, 5, 10, 20, and 50 μg/mL). dECM was solubilized in culture media and used to resuspend cells prior to plating. Plates were then centrifuged at 1500 rpm for 5 min and incubated at 37 °C and 5% *v*/*v* CO_2_. Spheroid (0, 5, 10, and 20 μg/mL) images were acquired using the Cytation 5 imaging system (Agilent Technologies, Santa Clara, CA, USA) in brightfield and RFP channels at 4× magnification. Spheroid area measurements were obtained through the Gen5 software (Agilent Technologies, Version 3.12). Images and measurements were collected at days 1, 2, and 7 post-seeding. At the endpoint, spheroids were collected for further downstream evaluation.

### 2.10. Spheroid Histological Evaluation

Spheroids (0, 10, 20, and 50 μg/mL) were transferred to 15 mm disposable base molds (Fisher Scientific, Hampton, NH, USA), gently washed with PBS, and then fixed with 4% paraformaldehyde (PFA) for 1 h. Following fixation, PFA was removed, spheroids were washed with PBS, and suspended in optimal cutting temperature (OCT) (Tissue-Tek O.T.C, Sakura Finetek USA, Torrance, CA, USA) compound. Embedded spheroids were snap frozen using liquid nitrogen and stored at −80 °C prior to cryosectioning.

### 2.11. RNA Sequencing

MDA-MB-231 cells were seeded with no dECM, 10 μg/mL, and 20 μg/mL of breast adipose dECM and tumor dECM in an AggreWell 400 24-well plate (Stem Cell Technologies, Vancouver, BC, Canada) that was prepped with Anti-Adherence solution (Stem Cell Technologies, Vancouver, BC, Canada), in accordance with the manufacturer’s protocol. After 4 days of culture, spheroids were collected for total RNA extraction using a Quick RNA Miniprep Kit (Zymo Research, Irvine, CA, USA), according to the manufacturer’s protocol. RNA sequencing was performed by Plasmidsaurus (Louisville, KY, USA). CPMs and DESeq2 [36,37] (Version 1.51.6, Benjamini–Hochberg false discovery rate method) were used to compare gene expression. Identification of significantly altered pathways was performed through the Enrichr gene set analysis web server [38,39], specifically from the Hallmarks database accessed in February 2026. All genes were considered significant if the adjusted *p*-value < 0.05. A |log2FC| > 1 was only applied for the volcano plot. Raw data files were uploaded to the NCBI’s Gene Expression Omnibus [40] (GEO Series accession #GSE328288). Reported data represent three independent experiments, and gene expression is provided in Supplemental File S1.

### 2.12. Statistical Analysis

Statistical analyses were performed using GraphPad Prism (v11.0.1). Two-way ANOVA, with Dunnett’s multiple comparisons, was performed to analyze differences in spheroid area, and one-way ANOVA with multiple comparisons and Welch’s unpaired *t*-tests were performed for ELISA assays and DNA quantification. *p*-values < 0.05 were considered statistically significant.

Generative artificial intelligence (GenAI) was not used in the design or writing of this study.

## 3. Results and Discussion

### 3.1. Decellularization of Breast Adipose Tissue Significantly Reduces Cellular DNA While Retaining Native Architecture and ECM

**Figure 1:**
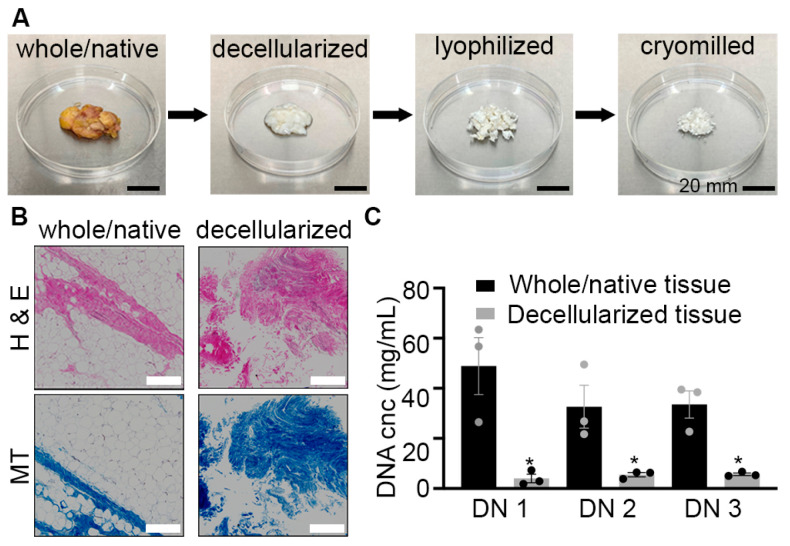
Decellularization of breast adipose tissue removed native cellular material while maintaining ECM protein networks. (**A**) The decellularization process started with whole/native tissue, decellularization to remove cellular material, followed by lyophilization to remove water, and finished with cryomilling to form a dECM powder. (**B**) Histological evaluation (hematoxylin and eosin, and Masson’s trichrome) of native and decellularized tissue confirms the removal of cellular material while maintaining the ECM. Images are representative samples of tissues evaluated. White scale bars = 300 µm. (**C**) DNA content of decellularized and native breast tissue as determined by nanodrop. Values represent the mean ± SEM from independent biological replicates. * *p* < 0.05.

We have previously demonstrated successful decellularization of triple-negative tumors [41], TNBC patient-derived xenograft (PDX) [33], and cell line-derived TNBC xenograft tumors [29]; and demonstrated differences in adipose tissue mechanical stiffness based on tissue location (proximal and distal to tumor site) [33]. Here, we sought to expand on our methods of tissue decellularization with a focus on the biochemical signaling induced by native ECM of breast tissue and tumors. Breast adipose tissue was decellularized and validated for loss of cell content and retention of ECM (Figure 1). Compositional analysis of the biochemical components lost or retained during the decellularization process demonstrated an increase in water content (28.5% ± 6.17 to 93.5% ± 0.17), complete removal of lipid content (46.17% ± 8.16 to 0% ± 0.0), and a decrease in protein content (25.33% ± 2.00 to 6.5% ± 0.17) (Figure S1). These findings are consistent with effective removal of adipocyte-associated components while retaining ECM structure (Figure S1) [42,43,44]. Histological analysis confirmed removal of cells with decellularization (H&E) in addition to preservation of ECM as observed through Masson’s trichrome staining for collagen fibers (Figure 1B). Removal of cell content was further confirmed through genomic DNA quantification, where decellularized breast tissue had a significant reduction in gDNA content, compared to native controls (Figure 1C). DNA gel electrophoresis further confirmed loss of DNA in decellularized adipose (Figure S2). The validation of the TNBC tumor (Tu-BcX-4IC PDX) decellularization is described in prior published works [33]. Through lyophilization and cryo-milling, we processed the decellularized breast tissue and PDX tumors from intact, spongy material into a dried powder (Figure 1A).

**Figure 2:**
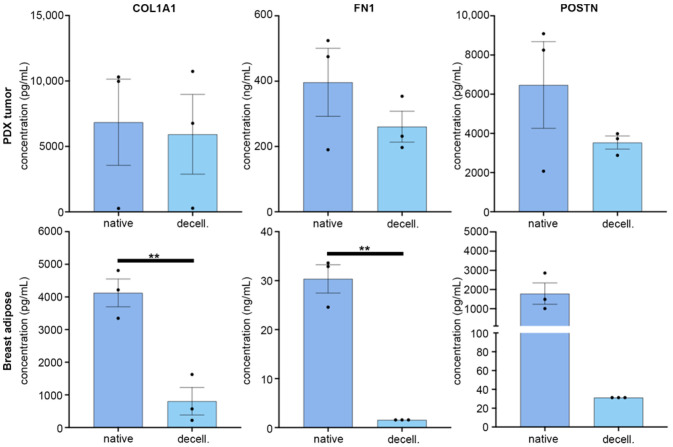
Validation of ECM protein expression within dECM tissues. Quantification of COL1A1, FN1, and POSTN protein expression in native and decellularized PDX and breast adipose tissue via immunoassay. All concentrations were normalized to concentration per 1000 µg/mL of protein loaded. Undetected proteins were reported with a value of LOD/2. Significance was determined using the unpaired Welch’s *t*-test (** *p* < 0.001).

Development of consistent decellularization of human tissue is critical for the development of in vitro models, with optimization to maximize retention of all native ECM proteins of critical importance. Prior studies have shown a loss of proteins altering gelation kinetics, limiting biomaterial applications [45], and is species/tissue-specific [46]. Maximizing ECM retention ultimately enables downstream applications for cell–matrix interactions, facilitating the development of biomimetic 3D tumor models [47]. Therefore, following cryo-milling, protein quantification for key ECMs of interest (POSTN, FN1, COL1A1) further confirmed the retention of ECM and demonstrated compositional differences between breast tissue dECM and tumor dECM (Figure 2). Here, we show an overall loss of ECM in the breast tissue dECM; however, tumor dECM retains native ECM proteins (COL1A1, FN1, and POSTN). While decellularization for adipose tissues showed an overall reduction in protein mass, likely due to lipid and cellular removal, we confirmed an enrichment of key ECMs of interest in tumor dECM. Compared to adipose tissues, the PDX tumor showed sample variability across biological replicates, implicating tumorigenesis results in ECM heterogeneity not seen in adipose tissue samples from different donors. Following confirmation of ECM content, adipose dECM was next evaluated for optimal dECM and cell concentrations to develop dECM 3D TNBC spheroids. Due to limited sample volume, tumor dECM was not used for seeding optimization and was retained for transcriptomic studies.

### 3.2. Incorporation of dECM Does Not Inhibit TNBC Spheroid Formation

**Figure 3:**
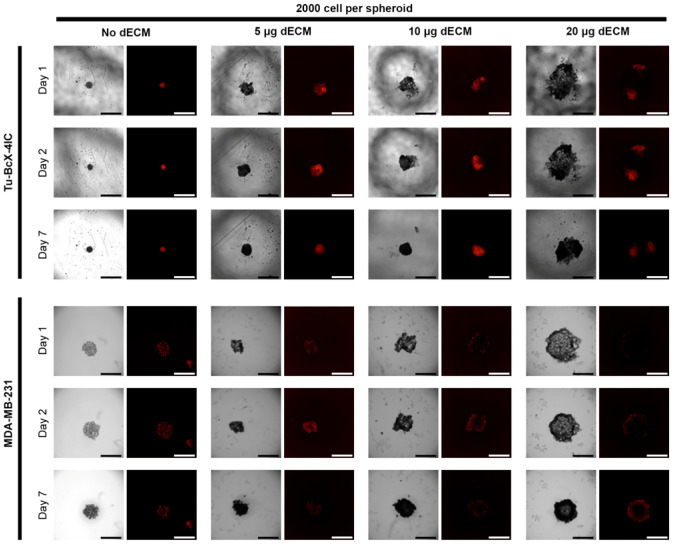
Increasing additions of dECM alter TNBC spheroid shape and size. Spheroids were seeded in U-bottom plates with increasing concentrations of breast tissue dECM (0, 5, 10, and 20 μg/mL), centrifuged, and incubated. Brightfield and RFP images were acquired on days 1, 2, and 7 post-seeding. Images are representative of samples of spheroids evaluated. Images are at 4× and scale bar = 2 mm.

TNBC cell lines (MDA-MB-231 and Tu-BcX-4IC) constitutively expressing a red fluorescent protein (RFP) were used to generate spheroids (2 k, 3 k, and 5 k cells per spheroid) with increasing amounts of breast tissue dECM (0, 5, 10, and 20 µg/mL dECM). Seeding density and dECM concentration were compared to determine the optimal conditions for spheroid formation and ECM organization. Control spheroids (0 µg/mL dECM) for all seeding densities self-assembled within 24 h without centrifugation, whereas dECM-containing groups were centrifuged at 1500 RPM for 5 min to facilitate aggregation. Spheroids (2 k) were cultured and imaged (brightfield and RFP) over 7 days to evaluate changes in spheroid properties (Figure 3). At 10 and 20 µg/mL, RFP imaging revealed elongated or segmented sphere formation infiltrated with dECM for Tu-BcX-4IC spheroids. After 7 days, only the 20 µg/mL spheroids maintained two separate cell populations separated by dECM; this trend was observed for all cell seeding densities (Figure 3 and Figure S3). The 2 k cells/spheroid MDA-MB-231s spheroids were larger and less compact over the 7 days, compared to the Tu-BcX-4IC spheroids (Figure S3). Addition of dECM resulted in an enrichment of cells around the edges of the spheroids (RFP imaging), suggesting that the center contained dECM. 3 k and 5 k TNBC spheroids exhibited similar effects with the incorporation of dECM (Figures S3 and S4). Increasing cell density resulted in larger spheroids, more variability with compaction, and uneven distribution of cells (MDA-MB-231 and Tu-BcX-4IC) within the dECM. As MDA-MB-231 spheroids were initially less compact, the RFP signal may be weaker due to lower cell compaction. Additionally, the increased incorporation of dECM may block the RFP signal as it is integrated within the spheroids, increasing overall size, cell distribution, and segmentation. These findings are in accordance with other reports where spheroid formation, using porcine dECM, resulted in decreased compaction with increased dECM concentration and the development of a necrotic core [35]. To quantify changes in spheroid formation, the spheroid area was calculated using the plate reader (Cytation 5) surface area function.

### 3.3. dECM Significantly Alters Spheroid Size

**Figure 4:**
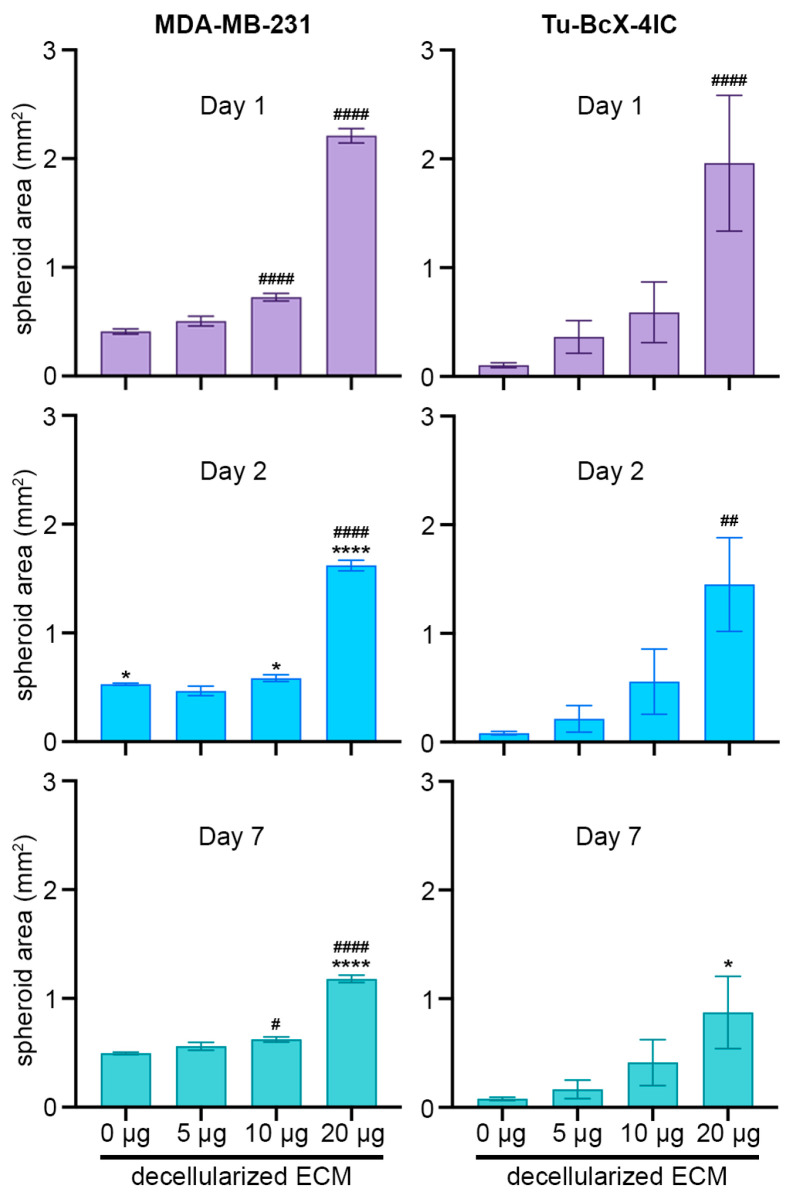
Increasing concentrations of dECM significantly increase TNBC spheroid size. Spheroid formation was evaluated by measuring spheroid area for both TNBC cell lines (2 k/spheroid) and for 0, 5, 10, and 20 µg/mL of dECM. Samples are mean ± SEM from independent biological replicates. Two-way ANOVA with Dunnett’s multiple comparisons was performed. * *p* < 0.05 and **** *p* < 0.0001 for each dECM concentration across the study duration, compared to Day 1 spheroid area. # *p* < 0.05, ## *p* < 0.01, and #### *p* < 0.0001 for each study sampling point (days), compared to 0 µg/mL of dECM.

Spheroid images were analyzed, and the average spheroid area for cell seeding density is reported in Figure 4 (2 k), Figure S5 (3 k), and Figure S6 (5 k). For all cell densities, spheroid size remained similar between the lower dECM concentrations (0 and 5 µg/mL) (Figure 4, Supplemental Figure S4). At higher dECM concentrations (10 and 20 µg/mL), a significant reduction in area was observed as the spheroids were allowed to grow in culture, compared to the Day 1 surface area. These trends were present for both TNBC cell lines, with the MDA-MB-231 cell line having less variability between biological replicates. As expected, spheroid size and variability increased with cell seeding density, with 5 k spheroids exhibiting the largest average area across all dECM concentrations (Figure S5). Of the combinations evaluated, 20 µg/mL dECM resulted in consistently significant changes in area over the study duration, with total area decreasing with time, suggesting compaction of cells with the dECM. Despite the observed compaction, 20 µg/mL TNBC spheroids were the only concentration that resulted in spheroids >1 mm^2^, after 7 days. In summary, the higher dECM concentrations (10 and 20 µg/mL) resulted in significant deviations in spheroid size when observing a single dECM concentration over time (*) and/or comparing dECM concentrations at a single time point (#). Based on spheroid formation observations and area analysis, we pursued 2 k spheroids for subsequent analysis of cell and ECM organization within the spheroid with Masson’s trichrome and H&E.

### 3.4. dECM Concentration Modifies TNBC Spheroid Organization

**Figure 5:**
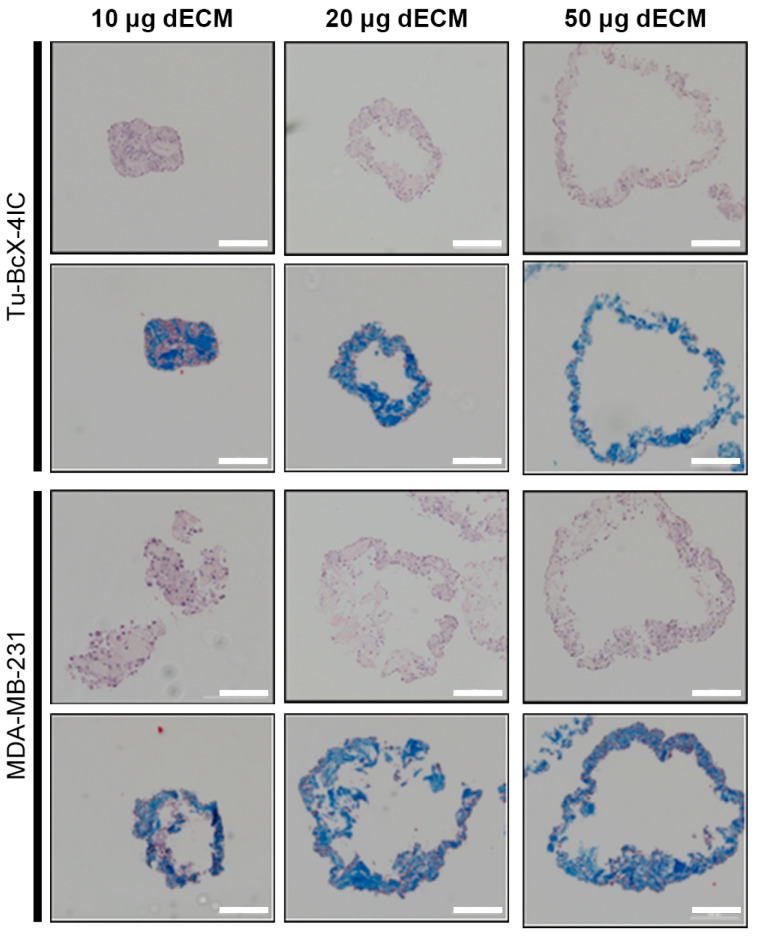
dECM directs cell and ECM organization in a concentration-dependent manner. TNBC spheroids were formed at 2 k cells/spheroid with dECM, centrifuged, and cultured for 7 days. After 7 days, spheroids were washed with PBS, fixed with PFA, washed with PBS, and suspended in OCT compound. Embedded spheroids were sent to the Tulane histology core for cryosectioning and staining (H&E and MTC). Images are representative samples of spheroids evaluated. Scale bar = 300 µm.

Fluorescent and brightfield imaging showed an increase in TNBC spheroid size and organization with increasing amounts of dECM. H&E and Masson’s staining confirmed that increased concentrations of dECM altered cell and ECM organization (Figure 5). Tu-BcX-4IC spheroids maintained a compact, spherical shape for low dECM concentrations (10 µg/mL) with cells and ECM tightly integrated (Figure 5), as seen with RFP imaging (Figure 3). With elevated concentrations (20 and 50 µg/mL), spheroids began to increase in size with ECM concentrated; however, increased dECM concentration was associated with loss of visible TNBC cells and ECM in spheroid centers. MDA-MB-231 cells exhibited reorganization of cells and ECM at the lowest concentration (10 µg/mL), with an increase in spheroid size and an empty/unstained central region. The addition of higher dECM concentrations (20 and 50 µg/mL) resulted in an increase in spheroid size and maintained the central empty region. Overall spheroid size, through H&E, demonstrated that MDA-MB-231 spheroids were larger than the Tu-BcX-4IC spheroids at higher concentrations (10 and 20 µg/mL). Use of 50 µg/mL of dECM resulted in similarly sized TNBC spheroids. The incorporation of decellularized tissue into spheroid modeling offers advantages as they retain ECM components, including a variety of structural proteins and signaling factors, which collectively play a critical role in cell behavior and tissue organization [19]. To determine the effect that dECM has on global transcriptomic changes and whether dECM composition alters the TNBC transcriptome, spheroids were generated without dECM, breast tissue dECM, or tumor PDX dECM and analyzed using RNA-sequencing.

### 3.5. Impact of dECM on the TNBC Transcriptome

**Figure 6:**
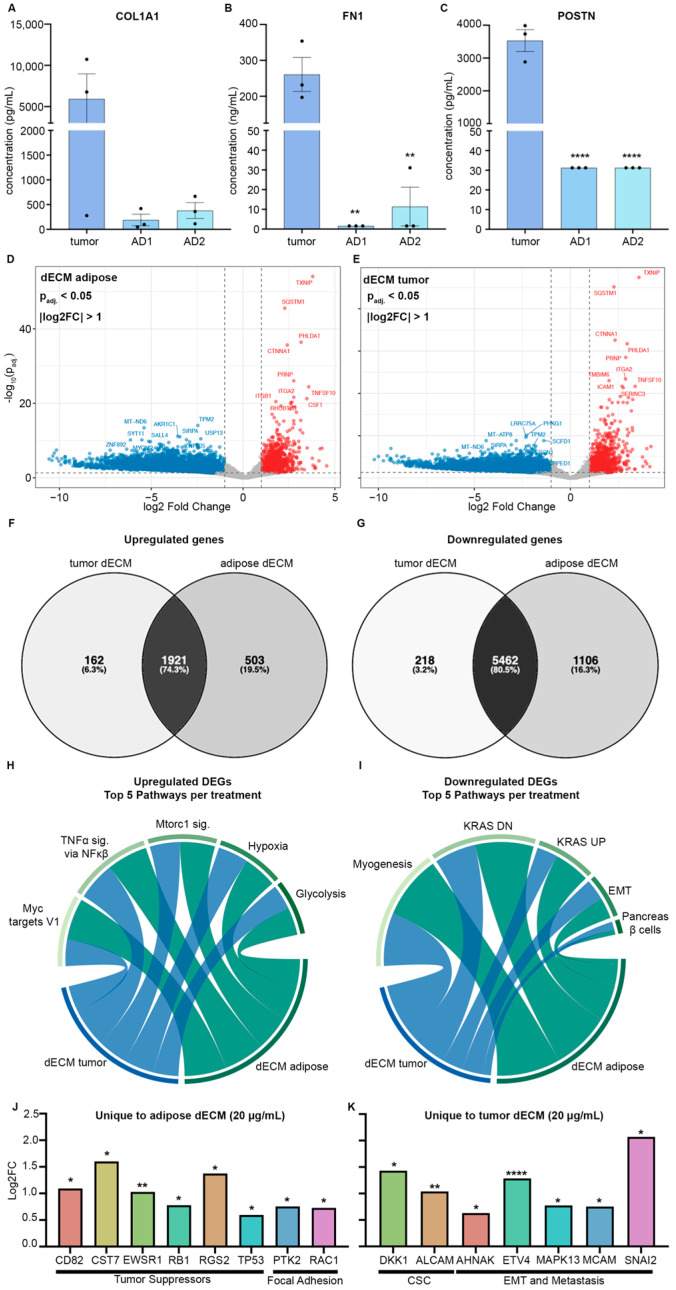
Differences in retained adipose and tumor dECM proteins elicit unique transcriptome expression changes. Quantification of COL1A1 (**A**), FN1 (**B**), and POSTN (**C**) protein expression in decellularized Tu-BcX-4IC PDX and breast adipose tissues (adipose donors (ADs) 1 and 2). All concentrations were normalized to concentration per 1000 µg/mL of protein loaded. Undetected proteins were reported with a value of LOD/2. Volcano plots for adipose (**D**) and PDX (**E**) 20 µg/mL dECM illustrate an induction of similarly significant (*p* < 0.05) up- (red) and downregulated (blue) genes. Venn diagrams depicting the overlapping, significantly upregulated (**F**) and downregulated (**G**) genes between tumor and adipose dECM (20 µg/mL). Chord plots show similar top pathway activations for up (**H**) and down (**I**) regulated genes. Differences in significantly changed genes that are unique to adipose (**J**) and tumor (**K**) dECM indicate differences in activation pathways. Values represent mean ± SEM from three independent biological replicates. * *p* < 0.05, ** *p* < 0.01, and **** *p* < 0.0001.

Comparisons of breast tissue (AD1 and AD2) and tumor PDX dECM demonstrated that tumor PDX dECM had increased expression of COL1 (Figure 6A), FN1 (Figure 6B), and POSTN (Figure 6C), compared to breast tissue dECM. These ECMs were chosen based on prior evaluations of the compositional differences in primary TNBC tumors and breast tissues [33,48,49]. COL1 expression for tumor dECM exhibited high biological replicate variability, also shown in Figure 2, resulting in no significant difference in ECM expression, compared to adipose donor dECM. Both FN1 and POSTN exhibited a significant decrease in expression in adipose dECM when compared to tumor dECM. Expression of FN1 and POSTN in tumor dECM was more homogenous across biological samples, when compared to COL1.

To determine global changes induced by dECM vs. spheroids formed without dECM, dECM MDA-MB-231 spheroids were grown in high (20 µg/mL) and low (10 µg/mL) breast tissue or tumor PDX dECM for 4 days. This time point was chosen to determine early transcriptional changes induced by dECM. Prior work by us has demonstrated that ECM-induced changes occur after at least three days in culture [48]. At the endpoint, total RNA was extracted, RNA sequencing was performed, and a comprehensive list of all significant (*p* < 0.05) genes is presented in Table S1. Results demonstrated that the addition of dECM (10 and 20 µg/mL) altered the breast cancer transcriptome, and both breast tissue and tumor PDX dECM had a similar overall impact on the TNBC transcriptome (Figure 6B and Figure S7). Breast tissue and tumor dECM comparably enhanced 1921 and repressed 5462 genes (Figure 6C). As expected, dECM elevated genes associated with cell–cell adhesion (CD44, ICAM), cell–ECM extracellular receptors (BCAR1, ITGA2, ITGA3, ITGA6, ITGB1, ITGB3, ITGB5), and intracellular signal transducers of these interactions (ACTN1, ACTN2, ILK, CTNNA1, PAK2, PXN, RAC2, RHOA, VCL, YAP1) (Table S1). Pathway analysis revealed that commonly upregulated genes were enriched in signaling hubs associated with glycolysis/cell metabolism, TNF/NFkB signaling, and mTOR signaling (Figure 6D). Of note, these signaling hubs had enhanced expression of genes in the AKT/mTOR, MAPK, and TNF/NFkB signaling cascades. Notably, downstream transcription factors, which are integral effectors of these pathways (ATF2, ELK1, ETS1, MYC, RELA), were upregulated. The addition of dECM induced downregulation of genes associated with broader signaling hubs that mediated EMT and growth factor signaling (Figure 6D) and expression of major collagen fibers (COL1A2, COL3A1, COL5A2). Prior works demonstrate that major fiber collagens I and III are repressed in triple-negative breast tumors compared to native breast tissue and hormone receptor-positive breast tumors [33,48,49]. Genes associated with WNT (canonical and non-canonical), BMP, and FGF signaling cascades were suppressed, suggesting alterations in EMT and growth factor-mediated signaling pathways. Interestingly, dECM decreased JNK and p38-associated MAPKs but increased key signaling components of the MAPK-ERK1/2 cascade, specifically MAPK1. Further, expression of DNA damage repair genes BRCA1 and RAD1 was downregulated in TNBC cells seeded in the tumor and breast adipose dECM. Prior works have demonstrated that ECM stiffness regulates genomic instability in cancer [50], and during mammary development, glycoproteins such as laminin regulate Brca1 in mouse-derived lines [51]. The role of ECM composition and genomic stability in TNBC has not yet been demonstrated.

Taken together, the addition of dECM to 3D TNBC spheroids enhanced pro-survival pathways, including AKT, NFkB, and ERK1/2, and repressed WNT-mediated ligand signaling. Integrin and FAK-mediated activation of WNT signaling in a WNT-independent manner has been demonstrated by others [52]. These gene changes are in line with our transcriptomic data. While additional studies are needed (e.g., expanding TNBC tumor tissue lines), it could be speculated that the observed loss of WNT ligands in cells seeded in dECM may be due to the release of reliance on these factors to activate WNT signaling [52]. Evaluation of distinct gene signatures in TNBC cells seeded in 20 mg/mL tumor PDX dECM compared to breast tissue dECM revealed tumor PDX dECM regulated 380 (162 up and 218 down) genes that were distinct from breast tissue dECM. In contrast, breast tissue dECM uniquely regulated 1609 genes (503 up and 1106 down) (Figure 6C). Tumor dECM induced genes associated with a cancer stem phenotype, EMT, and pro-metastasis, while adipose tissue dECM induced genes associated with tumor suppressors and focal adhesion (Figure 6E). The 10 mg/mL dECM demonstrated similar percentages of shared and distinct genes between tumor and adipose dECM (Figure S7). Similarly, to 20 mg/mL, 10 mg/mL dECM for both tumor and adipose regulated pathways associated with proliferation and cell survival (Figure S7F). In contrast to 20 mg/mL, unique genes for both tumor and adipose dECM were enriched for EMT, metastasis, and CSC phenotype (Figure S7G). Suggesting that ECM adhesion and composition participate in gene regulation.

Incorporation of dECM introduces cancer cells (MDA-MB-231) to more accurate in vivo conditions, influencing a variety of cellular pathways including adhesion, migration, invasion, and differentiation. Moreover, decellularized tissues offer spatial organization and architectural cues that guide cellular behavior, which scaffold-free cancer models cannot emulate [19]. Therefore, creating more biologically relevant tumor models requires the recapitulation of the TME. Incorporating decellularized tissue with 3D spheroid modeling enables researchers to more accurately mimic the native TME, which is pivotal for systematic evaluation of tumor development [8]. A limitation of this study was that the impact of dECM was evaluated in a single TNBC cell line (MDA-MB-231). and not reproducible in other lines (e.g., Tu-BcX-4IC). Future work will expand to other TNBC and breast cancer lines to fully explore the impact of dECM on the breast cancer transcriptome.

## 4. Conclusions

The recent paradigm shifts away from animal models to new methodologies have led to an increased interest in developing benchtop models that better capture the native TME. With current 3D models minimizing the importance of including native ECM, cell–ECM-driven changes are absent. This proof-of-concept study aimed to evaluate the impact of dECM on the development and organization of 3D tumor spheroids. Decellularization of adipose tissue resulted in a significant reduction in DNA, lipid content, and key ECM proteins (COL1A1, FN1, and POSTN), while Tu-BcX-4IC PDX decellularization only maintained key ECM proteins. Incorporation of adipose dECM (20 µg/mL) significantly enhanced spheroid size for both MDA-MB-231 and Tu-BcX-4IC cells. Histological evaluation showed cellular and matrix reorganization with increasing spheroid size and dECM concentration. Preliminary findings suggest that transcriptional changes might be dependent on tissue source. Prior works by us and others have demonstrated that the ECM of breast tumors varies based on breast cancer subtype, with hormone-positive (HR+) tumors being enriched for fiber collagens and TNBC tumors having elevated levels of glycoproteins [33,48,49]. Further, the response of breast cancer cells to distinct ECM compositions can be subtype specific, where collagens induce proliferation in HR+ breast cancer cell lines and, in contrast, induce cell senescence in TNBC lines [48]. While this study highlighted distinct differences in ECM-specific transcriptomic effects, these results represent comparisons of a singular dECM tumor and a single TNBC cell line. These preliminary works demonstrate a proof of concept for the biochemical impact of ECM on breast cancer cells, and additional studies should be performed to demonstrate breast cancer subtype-specific responses to dECM. In addition, due to the differences in ECM composition across tumors, dECM from additional tumor subtypes should be profiled. Future work will shift to further model validation through drug resistance, evaluating metabolomic and lipidomic profiles, and expansion of patient tissues to understand dECM-induced changes. Further, while our prior published works highlight the stiffness of breast adipose and tumor following decellularization [29,53], these studies did not aim to recapitulate the native breast stiffness of breast adipose or tumor. Additional studies will also focus on the incorporation of mechanical differences between healthy adipose and tumor tissues to fully impart in vivo properties into in vitro models.

## Data Availability

All data are contained within this manuscript and the Supplemental Documents. The RNA sequencing data discussed in this publication have been deposited in NCBI’s Gene Expression Omnibus, accessible through the GEO Series accession number (In process).

## Supplementary Materials

The following supporting information can be downloaded at https://www.mdpi.com/article/10.3390/bioengineering13060712/s1: Table S1. Adipose tissue donor information used for decellularization and spheroid formation; Figure S1. Decellularization reduces lipid and protein while increasing water content; Figure S2. DNA gels confirm removal of DNA from decellularization of adipose tissues; Figure S3. Increasing additions of dECM alter TNBC spheroid shape and size (3 k); Figure S4. Increasing additions of dECM alter TNBC spheroid shape and size (5 k); Figure S5. Increasing concentrations of dECM significantly increase TNBC spheroid size (3 k); Figure S6. Increasing concentrations of dECM significantly increase TNBC spheroid size (5 k); Figure S7. Differences in retained adipose and tumor dECM proteins elicit unique transcriptome expression changes; Table S2. Selected significant (*p* < 0.05) genes of interest log2FC, from RNA-sequencing; File S1. Raw DESeq genes.

## Abbreviations

The following abbreviations are used in this manuscript:

AD	adipose donor
CSC	cancer stem cells
dECM	decellularized ECM
DMEM	Dulbecco’s Modified Eagle Medium
EMT	epithelial to mesenchymal transition
ECM	extracellular matrix
H&E	hematoxylin and eosin
HR+	hormone-positive
IPA	isopropyl alcohol
MTC	Masson’s trichrome
OCT	optimal cutting temperature
PDX	patient-derived xenograft
RFP	red fluorescent protein
3D	three-dimensional
TNBC	triple-negative breast cancer
TME	tumor microenvironment
