# Supplementary material for: Toward More Translational Tumor Models: Breast dECM-Based 3D Systems Capture Native Microenvironmental Cues
Source: Bioengineering (Basel). 2026 Jun 21;13(6):712. doi: 10.3390/bioengineering13060712 (PMC13295492; doi:10.3390/bioengineering13060712)
Supplement: Supplementary file 1 [file bioengineering-13-00712-s001.zip › bioengineering-4289279-supplementary.pdf]

Supplementary Information

Table S1. Adipose tissue donor information used for decellularization and spheroid formation.

ID	BMI	Age
DN 306	28.6	57
DN 307	33.8	49
DN 308	25.2	27
DN 392	30	31
DN 414	30.6	32
DN 415	32.6	24
DN 12_2025	32.88	23

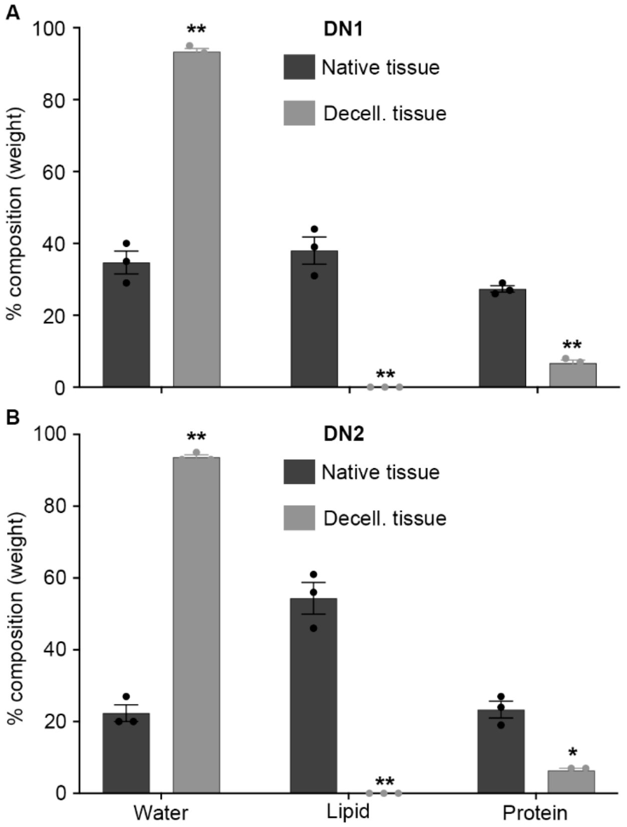

Figure S1. Decellularization reduces lipid and protein while increasing water content. Water, lipid, and protein content were evaluated before (native) and after (decell.) decellularization. Samples are mean ± SEM from independent biological replicates. Unpaired t-test was performed. *p<0.05 and **p<0.01.

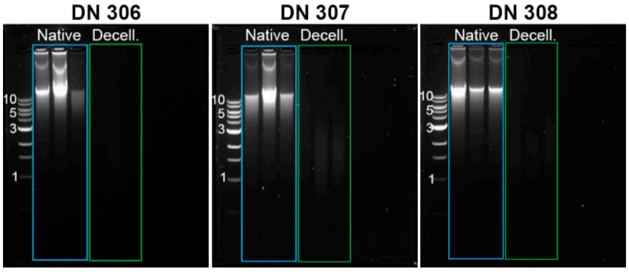

Figure S2. DNA gels confirm removal of DNA from decellularization of adipose tissues.

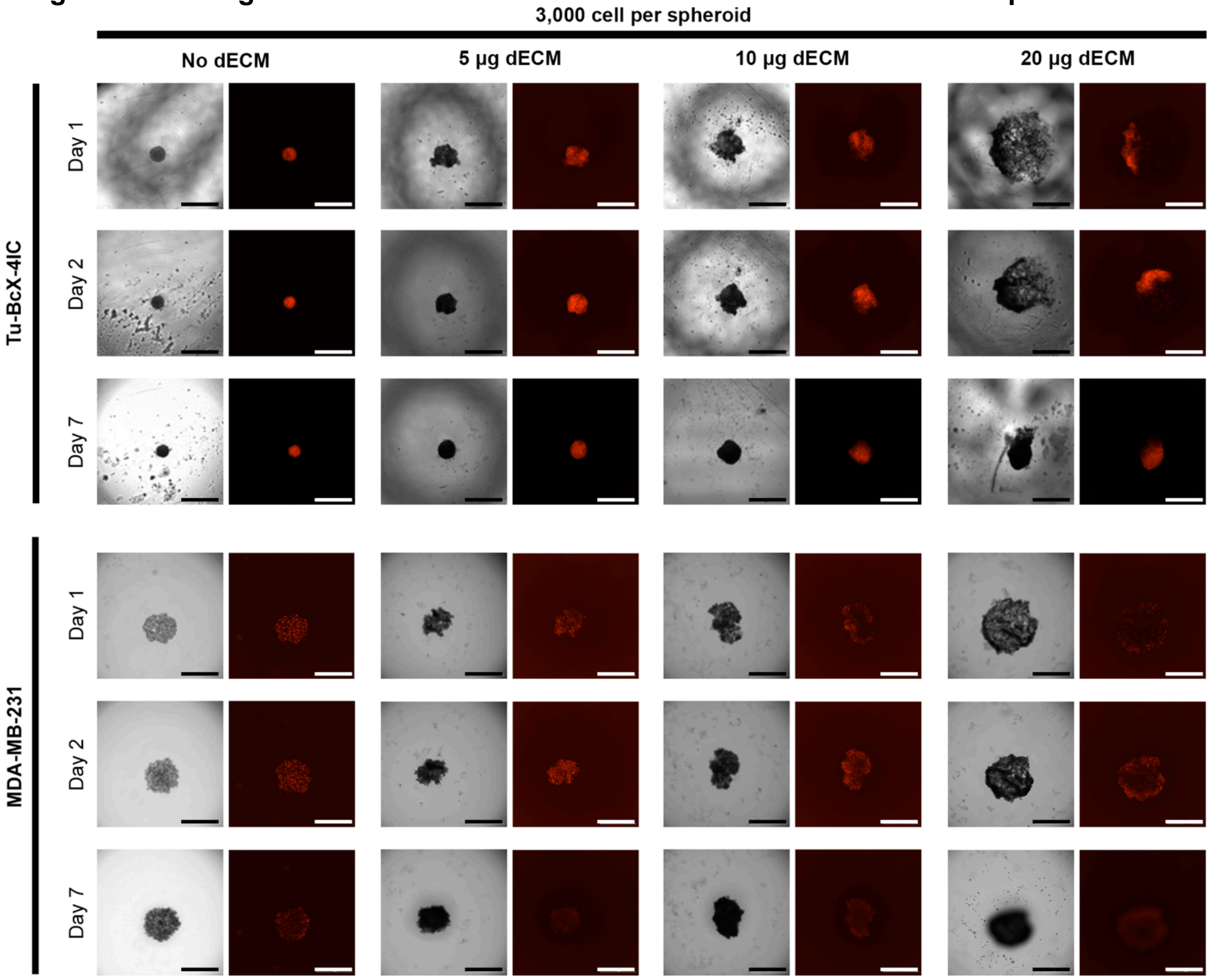

Figure S3. Increasing additions of dECM alters TNBC spheroid shape and size. Spheroids were seeded in U-bottom plates (3,000 cells/well) with increasing concentrations of breast tissue dECM (0, 5, 10, and 20 µg/mL), centrifuged, and incubated. Brightfield and RFP images were acquired at days 1, 2, and 7 post-seeding. Images are representative of samples of spheroids evaluated. Scale bar = 2 mm.

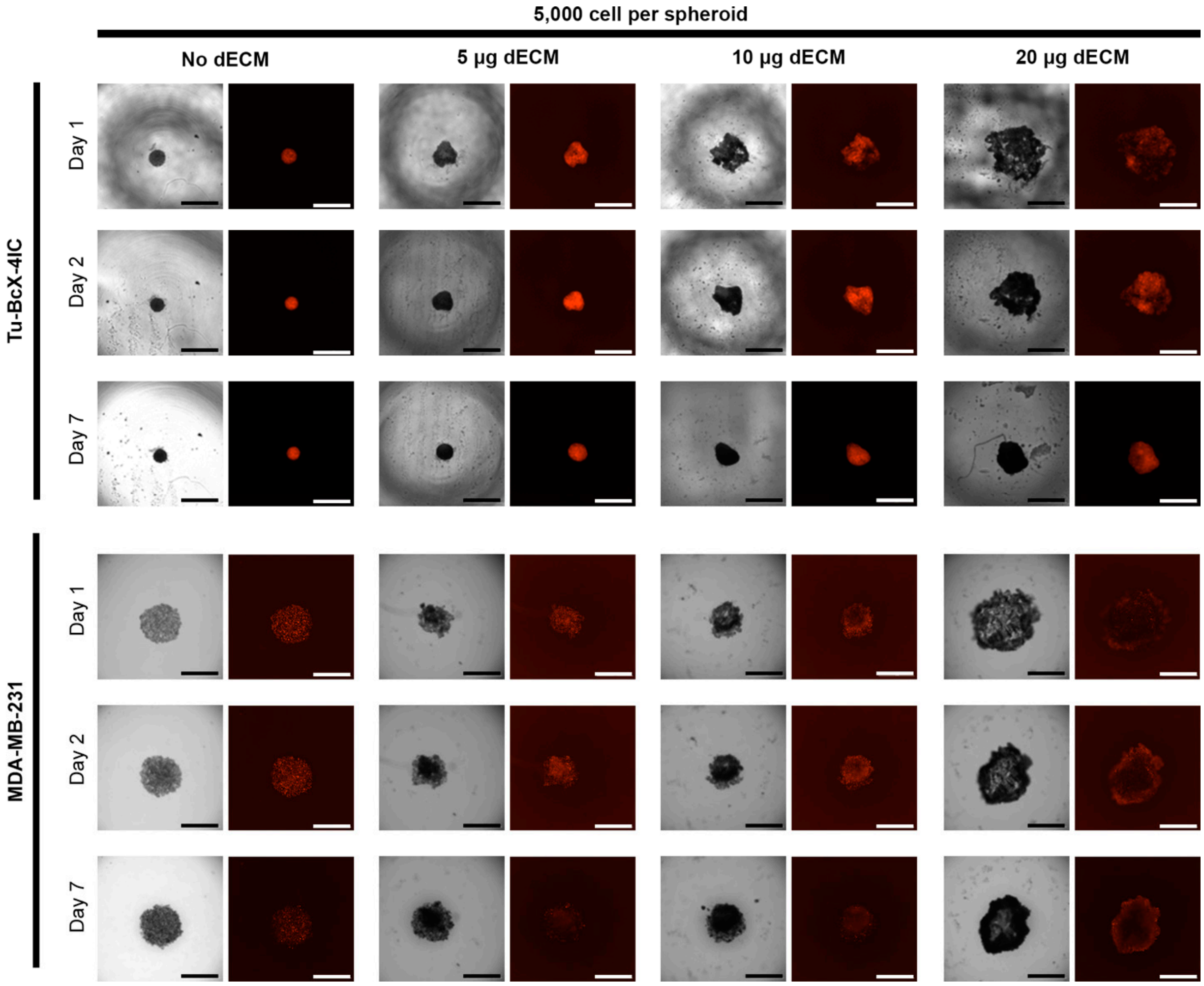

Figure S4. Increasing additions of dECM alters TNBC spheroid shape and size. Spheroids were seeded in U-bottom plates (5,000 cells/well) with increasing concentrations of breast tissue dECM (0, 5, 10, and 20 µg/mL), centrifuged, and incubated. Brightfield and RFP images were acquired at days 1, 2, and 7 post-seeding. Images are representative of samples of spheroids evaluated. Scale bar = 2 mm.

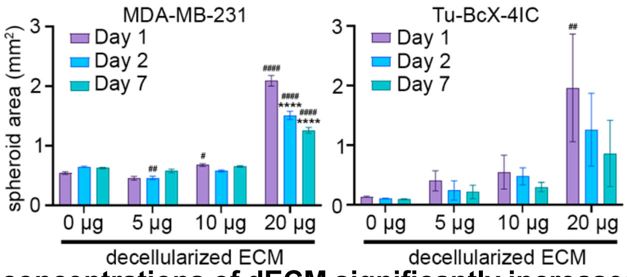

Figure S5. Increasing concentrations of dECM significantly increases TNBC spheroid size. Spheroid formation was evaluated by measuring spheroid area for both TNBC cell lines (3k/spheroid) and for 0, 5, 10, and 20 µg/mL of dECM. Samples are mean ± SEM from independent biological replicates. Two-way ANOVA was performed. ****$p<0.0001$ for each dECM concentration across the study duration, compared to Day 1 spheroid area. #$p<0.05$, ##$p<0.01$, and ####$p<0.0001$ for each study sampling point (days), compared to 0 µg/mL of dECM.

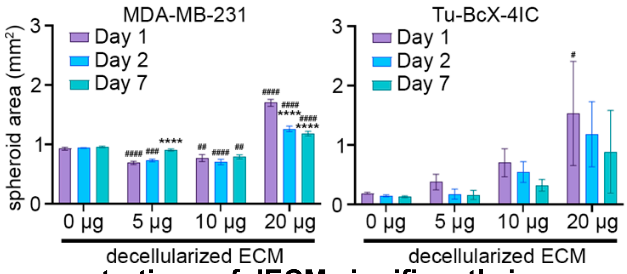

Figure S6. Increasing concentrations of dECM significantly increases TNBC spheroid size. Spheroid formation was evaluated by measuring spheroid area for both TNBC cell lines (5k/spheroid) and for 0, 5, 10, and 20 µg/mL of dECM. Samples are mean ± SEM from independent biological replicates. Two-way ANOVA was performed. ****p<0.0001 for each dECM concentration across the study duration, compared to Day 1 spheroid area. #p<0.05, ##p<0.01, ###p<0.001, and ####p<0.0001 for each study sampling point (days), compared to 0 µg/mL of dECM.

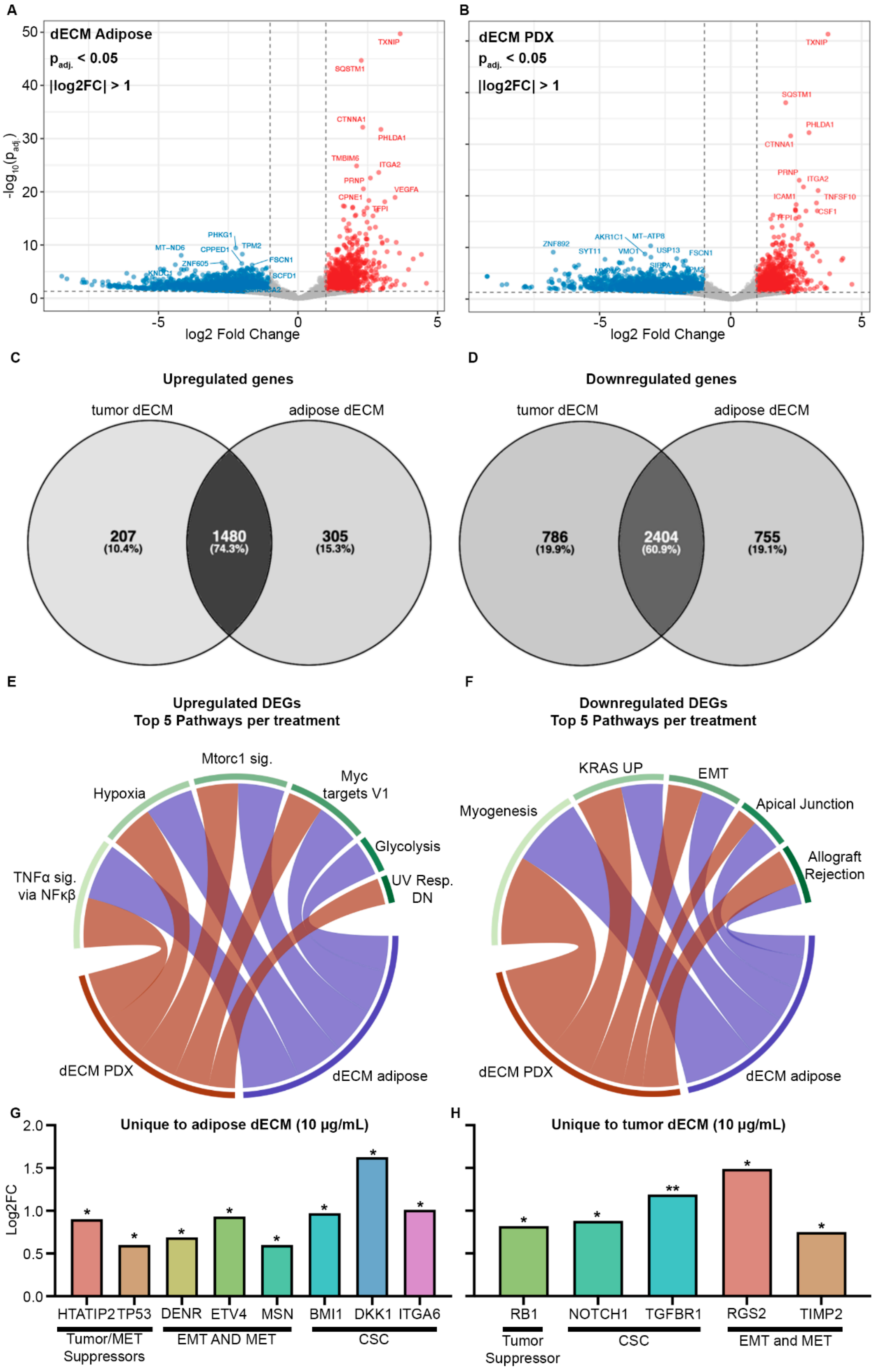

A
dECM Adipose
$p_{adj.}$ < 0.05
|log2FC| > 1
$-log_{10}(p_{adj})$
log2 Fold Change
TXNIP
SQSTM1
CTNNA1
PHLDA1
TMBIM6
ITGA2
PRNP
VEGFA
CPNE1
TFPI
PHKG1
TPM2
MT-ND6
CPPED1
FSCN1
ZNF605
SCFD1
B
dECM PDX
$p_{adj.}$ < 0.05
|log2FC| > 1
log2 Fold Change
TXNIP
SQSTM1
PHLDA1
CTNNA1
PRNP
ITGA2
ICAM1
TNFSF10
CSF1
TFPI
ZNF892
AKR1C1
MT-ATP8
SYT11
VMO1
USP13
FSCN1
C
Upregulated genes
tumor dECM
adipose dECM
207 (10.4%)
1480 (74.3%)
305 (15.3%)
D
Downregulated genes
tumor dECM
adipose dECM
786 (19.9%)
2404 (60.9%)
755 (19.1%)
E
Upregulated DEGs
Top 5 Pathways per treatment
Mtorc1 sig.
Hypoxia
Myc targets V1
Glycolysis
UV Resp. DN
TNFα sig. via NFκβ
dECM PDX
dECM adipose
F
Downregulated DEGs
Top 5 Pathways per treatment
KRAS UP
EMT
Myogenesis
Apical Junction
Allograft Rejection
dECM PDX
dECM adipose
G
Unique to adipose dECM (10 µg/mL)
Log2FC
HTATIP2
TP53
DENR
ETV4
MSN
BMI1
DKK1
ITGA6
Tumor/MET Suppressors
EMT AND MET
CSC
H
Unique to tumor dECM (10 µg/mL)
RB1
NOTCH1
TGFBR1
RGS2
TIMP2
Tumor Suppressor
CSC
EMT and MET

Figure S7. Differences in retained adipose and tumor dECM proteins elicits unique transcriptome expression changes. Volcano plots for adipose (**A**) and PDX (**B**) 10 µg/mL dECM illustrate an induction of similarly significant ($p<0.05$) up and downregulated genes. Venn diagrams depicting the overlapping, significantly upregulated (**C**) and downregulated (**D**) genes between tumor and adipose dECM (10 µg/mL). Chord plots show similar top pathway activations for up (**E**) and down (**F**) regulated genes. Differences in significantly changed genes that are unique to adipose (**G**) and tumor (**H**) dECM indicate differences in activation pathways.

Table S2. Selected significant ($p<0.05$) genes of interest log2FC, from RNA-sequencing.

Gene	Adipose 10	Adipose 20	Tumor 10	Tumor 20
ACTN1	1.946312882	1.97648846	1.903123012	2.18125368
ACTN4	0.73701388	0.903219098	0.544690694	1.033895398
AIFM1	-0.930319572	-0.954792206	-0.772801151	-0.879396755
AKT1	1.609251098	1.636094164	1.486313196	1.572895023
ARAF	1.500340282	1.524327737	1.234926813	1.659554129
ARHGAP5	2.044616769	2.018094211	1.998009846	2.141558264
ATF2	1.863642943	1.451333054	1.599782396	1.601047749
ATM	-1.048378978	-1.940226577	-1.072766753	-1.630485701
AXL	1.456027458	1.024408571	1.046980218	1.281632702
BAD	0.993904486	1.14341922	0.907037233	0.97806486
BCAR1	1.165614197	1.651865518	1.290770342	1.623869652
BCL10	2.236745498	1.728988801	1.872457278	1.793465979
BCL3	2.020433784	2.355832121	2.125704287	2.236883665
BIRC2	1.142828161	1.203435928	1.158260628	1.343854118
BMP2	-2.453849636	-4.76530498	-3.057815783	-3.964301516
BMP6	-1.802494915	-3.381236946	-2.531734654	-2.927390501
BMP7	-3.969970038	-5.413640483	-2.294463204	-3.560974546
BRAF	-0.817439225	-1.502062356	-1.071996501	-1.247030596
BRCA1	-1.825599594	-3.155321561	-1.471214017	-2.835566056
BTG2	-1.171383554	-1.897946706	-1.62557106	-2.141508608
CAB39	1.801980516	1.948370352	1.886150239	1.877219336
CAPN2	1.147170644	0.940669947	0.786316481	1.004291197
CASP8	1.652590213	1.365632317	1.492437765	1.657392276
CAV1	1.877293775	1.936012947	1.638924011	1.655409125
CCND2	-1.960391063	-3.384278712	-2.938965731	-3.230070771
CCND3	0.902340506	1.200842864	0.848714544	1.20620555
CD44	1.043778209	1.10988713	0.96467055	1.133793784
CDH1	-3.617037874	-4.945604478	-4.418639848	-4.028149114
CDH2	-1.691352898	-4.17372697	-1.537083549	-3.688537231
CDK4	1.865820837	1.747508016	1.492140698	1.755882639
CDKN1B	1.176514078	1.376845159	1.271126259	1.206776397

CDKN1C	-1.193991486	-1.252327501	-1.255482704	-1.36731845
CDKN2A	-2.215361047	-3.578209128	-3.422146493	-2.690197983
CFB	0.732592717	1.556079775	0.805140128	1.639613866
COL1A1	-1.843377379	-3.356766216	-1.657668596	-2.727108288
COL1A2	-2.215666238	-4.119007536	-2.101440943	-2.979214137
COL3A1	-2.929081056	-4.566961334	-3.711569624	-4.272271059
COL5A2	-2.877361302	-3.882191288	-2.78991726	-3.761598677
CSF3	1.530371173	2.067061552	1.438944045	2.087545357
CSNK2A1	-0.412573165	-0.786823252	-0.521204958	-0.678925632
CXCL8	2.706202812	2.600455264	2.516741228	2.177317144
CXXC4	-1.938692178	-2.321184903	-1.465265032	-2.259371885
DDIT4	1.92911604	2.144359252	1.934979296	1.840973448
DIAPH1	1.494415489	1.399494042	1.307970523	1.603109407
DIXDC1	-1.471831294	-2.896282288	-1.155735951	-2.502852525
DLK1	-0.676583022	-4.73345658	-0.758780884	-3.888432677
DMC1	-1.06515508	-4.772490303	-0.647039554	-2.104967513
DSC2	-1.450157759	-1.231965213	-1.349392176	-1.060134131
EGFR	1.577172119	1.68469346	1.60462403	1.564126642
EGR1	1.911774649	2.184768425	1.957356371	2.042180428
EIF4B	1.080027088	1.37805729	1.072561781	1.043363674
ELK1	1.07500535	1.228636608	1.088636379	1.062944857
ERBB3	-1.230703137	-2.556420897	-1.436762363	-2.224780967
ESR1	-0.571949464	-2.679980572	-0.380633452	-2.480412299
ETS1	1.672779027	1.244986834	1.445452725	1.356859358
FGF13	-2.990613322	-5.073858171	-2.896352346	-6.553445005
FGF14	-2.194486695	-2.594474915	-2.207982595	-1.373095703
FGF2	-0.690775996	-4.195575433	-1.250933871	-5.993352328
FGF22	-2.257650565	-4.21117154	-2.435038396	-2.666736947
FGF23	-0.982144208	-6.146589935	-0.770558627	-4.210881289
FGF5	-0.983506371	-1.408897573	-1.423351267	-1.573712557
FGF7	-0.723882468	-2.920959379	-0.4314826	-2.939758643
FGF9	-2.93276827	-5.788371688	-3.826787921	-5.105945488
FKBP1A	1.597030183	1.541144321	1.493351175	1.545198758
FKBP8	0.87100444	0.658914033	0.517414359	0.936872012
FLNA	0.701878399	0.690904242	0.538069722	0.924103475
FOXA2	1.278620086	1.315543263	1.148331583	1.317742959
FOXC2	-0.762163861	-4.742282639	-0.741728319	-2.427272261
FRZB	-3.757893935	-1.928610025	-2.063153749	-2.998460481
FZD3	-0.740800527	-1.653123323	-0.813081553	-1.335010821

FZD4	-1.351674414	-1.593810781	-1.291068262	-2.132495329
FZD7	3.957273214	3.771222041	3.777379657	3.760353697
FZD8	-1.409977189	-1.941090303	-2.238416904	-2.259453727
GADD45G	-2.680080797	-1.983815215	-1.974592675	-3.262217181
GDF11	-2.197506721	-2.60577987	-1.649413438	-2.370229714
GDNF	-0.741616096	-4.206880221	-1.197007826	-3.659278547
GSK3B	-0.838598687	-1.352008047	-1.061531665	-1.199415594
HDAC1	1.144107255	1.434475603	1.171518726	1.516036874
HUS1	-1.224098552	-1.579568006	-1.055311646	-1.500586206
ICAM1	2.361548663	2.601612359	2.50073905	2.693927278
ID1	1.693016846	2.331911936	2.227692201	1.967275313
IGF1	-1.075182576	-5.365353434	-0.761427823	-4.245959694
IGHMBP2	-1.167616815	-1.058875322	-0.850593697	-1.129850063
IL10	-2.247930018	-4.037845042	-2.131416156	-2.506927485
IL1A	3.072994381	2.609998907	2.551706409	2.272611609
IL1B	1.659894588	1.64150704	1.54058113	1.373649853
IL1RN	-0.66761736	-3.381438006	-1.183965514	-2.449563525
IL6	2.904824869	2.761288923	2.752305024	2.622027319
ILK	1.062285805	1.043358725	0.836315468	1.04724414
INHBA	-1.646571405	-2.642134693	-1.431798507	-2.548833128
INHBB	-0.440238896	-1.032811015	-0.751107705	-0.992124753
IP6K3	-1.704604969	-5.864419481	-3.371968104	-7.608197049
IRS1	1.583395476	1.91849184	1.846020798	2.007944903
ITGA1	1.586150303	1.823393896	1.797543611	2.1491865
ITGA2	2.890983871	2.762516791	2.778438179	2.876584651
ITGA3	0.905651675	0.748178109	0.712455356	1.01611448
ITGA6	1.012715604	1.125606159	0.901215263	1.208707302
ITGB1	1.663259269	1.78788822	1.612912878	1.631913383
ITGB3	2.041680776	2.232178369	2.264317639	2.371375138
ITGB5	0.888898796	1.249598816	1.118961451	1.022758876
JAG2	-1.507763577	-1.889787575	-2.028884291	-1.681174738
KRT14	-0.470033466	-5.348500429	-0.551035223	-4.991316401
KSR1	-1.696565376	-2.436272653	-2.227680153	-2.572711982
LATS1	1.718548236	1.566620753	1.518199991	1.672223058
LIG1	-1.117477913	-1.300179147	-1.167381039	-1.819095186
LMNA	1.017066782	1.220273445	0.960628698	1.188941355
LMNB2	1.036695781	1.55477803	1.247636416	1.284476512
MAP2K6	-1.49929026	-4.095768266	-1.58716341	-3.780062225
MAP2K7	0.589257479	1.061406978	0.839718923	0.962159815

MAP3K2	0.792525803	0.698515727	0.606008151	0.65641048
MAP4K1	-3.741007858	-4.709497806	-3.130897569	-2.758443186
MAPK1	1.196666468	1.380909013	1.178506627	1.384100533
MAPK10	-1.320119365	-4.969012282	-0.788273141	-4.041932166
MAPK8IP2	-2.259704218	-4.255281143	-2.876241641	-3.278758381
MAPKAP1	1.012502264	1.283594976	1.082448587	1.123051104
MAPKAPK2	1.216075227	1.178120125	1.057459313	1.399200981
MAX	1.113929429	1.096581338	1.255399822	1.193498388
MEF2C	-2.230946679	-2.645242857	-2.08836304	-2.350501271
MITF	-1.60545645	-2.185118505	-1.531494472	-2.303351442
MKNK1	-0.970321123	-1.414063941	-0.932382224	-1.402057009
MMP2	-2.276072072	-3.511436973	-3.602128012	-4.664798551
MMP3	-0.594441414	-2.335879913	-0.300057753	-2.314165919
MMP9	-1.863026469	-3.681010731	-2.210569759	-2.771596633
MSH2	-0.950278329	-1.07368521	-1.11470925	-0.872421768
MYC	2.408832263	1.669237317	2.172878734	2.0652019
N4BP2	-0.786492081	-1.659799059	-0.845965923	-1.492268467
NFATC4	-2.388300053	-4.14813711	-2.679857518	-2.823367114
NFKBIA	1.649940627	2.332015417	1.983971319	2.096617502
NGF	-1.195374252	-1.37110786	-0.856355354	-1.307625257
NKD1	-3.210907486	-6.196174006	-3.597493047	-4.800075062
NOTCH1	0.578893861	0.921150547	0.88129559	1.053436118
NOTCH2	1.342301195	1.478742868	1.389569862	1.725762572
NRAS	1.809000564	1.816363238	1.658832029	1.651544068
NRG1	-1.013395684	-1.359965026	-1.11192143	-1.456352312
NRG2	-1.239096027	-3.775479886	-0.840743744	-2.955990896
NRG3	-4.931684582	-7.575773764	-1.000618885	-3.607688928
OCLN	-1.120769938	-1.258591865	-1.006880701	-1.220272778
OGG1	-1.505220857	-1.968355663	-1.507167523	-1.869807587
PABPC1	0.985236718	0.672327155	0.505793233	0.883866328
PAK2	0.884329776	0.986224854	1.023397984	1.006901982
PITX2	-3.657290167	-2.365564939	-1.467251537	-2.03846758
PPM1A	0.966189792	0.863527595	0.996322693	0.969877693
PPP2CA	1.161004416	1.018631399	0.925828468	1.03862976
PRDX6	1.106573415	1.35113029	1.075724984	1.134988162
PXN	1.292581812	1.696732719	1.51549924	1.572945038
RAC2	1.099572951	1.61583637	1.371672664	1.042801845
RAD1	-0.770487112	-1.029195484	-0.813024692	-1.06403081
RAD51B	-1.690184354	-1.465721149	-0.78064149	-2.576823295

RAD9A	-1.999502908	-2.017612634	-1.97505851	-2.108102187
RAF1	-1.188483176	-1.047570792	-0.816663177	-1.391518204
RBL2	1.023576072	1.176949782	1.001586672	1.328803297
RELA	1.273399746	0.971126903	0.852395881	1.209000887
RHEB	1.599620685	1.200564866	1.170010134	1.137563295
RHOA	1.097932975	1.330901214	1.036857299	1.061444218
RRAGA	0.869773611	0.867068052	0.682226941	0.710370061
RRAGC	0.985447933	0.933882765	0.790806856	1.095607321
SAV1	1.096856316	1.127669012	1.072642852	0.939044003
SEMA4A	-2.12298166	-4.618779694	-1.236951911	-4.712367698
SFRP1	-2.509031724	-4.103213791	-0.810028282	-2.550771575
SFRP4	-0.541376128	-4.218137366	-0.816403566	-2.505527374
SHC1	1.170004232	1.330079944	1.15635232	1.169390577
SIRT1	1.981197626	1.633562471	1.562011456	1.502993776
SLC20A1	1.461792966	1.835422667	1.615697632	2.096353932
SPARC	-1.780586863	-3.851034601	-1.819271399	-2.657902012
SPP1	-2.04653782	-3.573209954	-1.982025672	-2.708992289
SRF	0.867804753	0.968617987	0.875776299	0.984899114
STAT3	0.608525275	0.768633314	0.622509409	0.795916167
STK11	0.679650354	0.989187033	0.798189816	0.836952807
STRADB	1.625037016	1.631056196	1.651698634	1.540298128
TCF4	-2.44025419	-5.589378473	-1.99446832	-4.754616659
TCF7	-0.645409066	-2.895516319	-0.855515835	-2.64749157
TGFB3	-2.106357878	-2.940337621	-2.507539406	-3.185345387
TGFBR1	0.800085833	1.176469318	1.193632107	1.355614216
TLE2	-2.028174774	-4.3406944	-3.172413284	-4.083235214
TMED4	0.713643859	0.805059775	0.690543351	0.62763384
TNFAIP3	1.493864014	1.212052827	1.070192845	1.207008836
TNFRSF10A	0.706901461	1.24152582	1.184676072	1.415338292
TNFRSF10B	1.219799015	1.464165692	1.30310529	1.30347167
TNFRSF10D	0.741308206	0.992411978	0.943349654	0.892090939
TNFRSF1A	0.473461317	0.856056985	0.761631002	0.859200168
TNFRSF21	1.663499918	1.932650639	1.67432479	2.136410754
TNFSF10	3.105610391	3.578519477	3.336278071	3.377746336
TOLLIP	0.647725668	0.835480576	0.617133831	0.812935455
TSPAN13	-2.361278298	-2.740611651	-2.214212157	-2.826766455
TWIST1	-3.027883588	-6.070594963	-3.912587733	-3.843509099
VCAN	-1.925835044	-4.181193854	-1.944776287	-3.729813138
VCL	1.137969198	1.079230402	0.925687244	1.082498864

VEGFA	3.475365652	3.140984901	3.307183633	3.17973757
VEGFB	0.699037552	0.730380445	0.613178153	0.855441755
VEGFC	3.183285467	3.134326705	3.077677884	2.802027337
VPS13A	-1.027919866	-1.388855388	-0.951611854	-0.784586905
WASL	1.453527476	1.478297146	1.18898331	1.536272873
WIF1	-2.477949276	-4.993723683	-4.523783908	-3.537587403
WNT2	-0.425659121	-0.684107106	-0.523123581	-0.636819919
WNT2B	-1.053654885	-3.233694227	-1.071093393	-2.680281309
WNT3A	-0.549436984	-1.050394113	-0.703574535	-0.93433659
WNT4	-1.416628946	-3.348891103	-2.374845463	-2.202588162
WNT5A	-2.151701622	-2.958541524	-2.982908888	-2.551906788
XRCC2	-1.888359119	-2.68812186	-1.887913513	-2.150582315
XRCC3	-1.250721985	-2.014249075	-1.914515784	-2.059591853
YAP1	1.880415773	1.713733566	1.683981403	1.589900065
YWHAQ	1.519773311	1.371713909	1.184805068	1.337257059
ZEB2	-1.678926148	-2.268502512	-1.49815734	-1.897414132
